# Corona Pandemic: Assisted Isolation and Care to Protect Vulnerable Populations May Allow Us to Shorten the Universal Lock-Down and Gradually Re-open Society

**DOI:** 10.3389/fpubh.2020.562901

**Published:** 2020-09-30

**Authors:** Johnny Ludvigsson, Matthias G. von Herrath, Roberto Mallone, Karsten Buschard, Corrado Cilio, Maria Craig, Jorma Ilonen, David Leslie, Julie E. M. McGeoch, Darius Schneider, Jay S. Skyler, Malin Flodström Tullberg, Didier Hober

**Affiliations:** ^1^Department of Biomedical and Clinical Sciences, Crown Princess Victoria Children's Hospital and Division of Pediatrics, Linköping University, Linköping, Sweden; ^2^La Jolla Institute for Allergy and Immunology, La Jolla, CA, United States; ^3^Université de Paris, Institut Cochin, CNRS, INSERM; Assistance Publique Hôpitaux de Paris, Hôpitaux Universitaires de Paris Centre-Université de Paris, Cochin Hospital, Service de Diabétologie et Immunologie Clinique, Paris, France; ^4^Bartholin Institute, Rigshospitalet, Copenhagen, Denmark; ^5^ImmunoVirology Unit, Department of Clinical Sciences, Lund University, Lund, Sweden; ^6^Children's Hospital at Westmead and University of Sydney, Sydney, NSW, Australia; ^7^Immunogenetics Laboratory, Institute of Biomedicine, University of Turku, Turku, Finland; ^8^Blizard Institute, Queen Mary, University of London, Whitechapel, United Kingdom; ^9^Department of Molecular and Cellular Biology, Harvard University, Cambridge, MA, United States; ^10^Department of Medicine, Diabetes Research Institute, University of Miami, Miami, FL, United States; ^11^Department of Medicine Huddinge, The Center for Infectious Medicine, Karolinska Institutet, Karolinska University Hospital, Stockholm, Sweden; ^12^Université de Lille, CHU Lille, Laboratoire de Virologie-ULR3610, Lille, France

**Keywords:** COVID19, public health, economic depression, vulnerable groups, isolation, corona

## Introduction

Severe Acute Respiratory Syndrome Coronavirus-2 (SARS-CoV-2), the virus that causes coronavirus disease 2019 (COVID-19), has emerged as a major threat to mankind. The proportion of those dying from COVID-19 is highest in the elderly population and those with pre-existing co-morbidities (such as severe obesity, hypertension, diabetes, cancer, chronic respiratory, renal, or cardiovascular disease) (1–4). As individuals can spread the virus rapidly without exhibiting any symptoms, multiple countries have taken steps to shut down schools, universities, whole companies and businesses, and entire villages, towns, and countries have been isolated. These drastic measures will have enormous economic, public health, and psychological consequences (5–9).

It is likely that we are significantly underestimating the prevalence of COVID-19. The proportion of asymptomatic infected individuals has been estimated between 17.9% (10) and 51% (11), but could be as high as 80% (12). It has not been established whether transmission can occur before symptoms appear, but the virus has been detected in the stool of an asymptomatic child (13) and there are indications of transmission from asymptomatic carriers (14). In some countries, it may be too late to sufficiently “flatten the curve” (15) based on a universal lock-down strategy. Coercive measures could be counterproductive and erode public trust and cooperation (16). Moreover, it is of great concern that large-scale lock-down of society has many additional negative impacts. It is of critical importance therefore that more refined strategies are considered, which may help containing the pandemic whilst minimizing significant societal disruption, and help allocate resources in the most effective ways. Others have pointed out that, despite the breadth and allure of travel bans and mandatory quarantine, an effective response to SARS-CoV-2 requires newer, more creative legal tools but without clear recommendations on how to achieve this (17). While certain countries start to re-open their societies and borders, most remain on lockdown measures to different extents. We suggest here that more selective assisted isolation of vulnerable populations would reduce the predictable increase in hospital admissions and more rapidly alleviate the fallout from total lockdown measures.

## Alternative Approach to Complete Lock-Down

SARS-CoV-2 infection rarely leads to symptoms in people below 20 years of age (18) and usually causes mild symptoms in people up to the age of 50 years (19). Additional risk factors (2, 4, 20) negatively impact the outcome, and may potentially include high dose exposure in health care settings. Even though COVID19 sometimes leads to need for treatment at intensive care units (ICU) also for younger individuals, the virus appears most dangerous for a selected group of the most vulnerable people. In several countries, the average age of the deceased patients is around or above 80 years. We must consider diverting our major efforts to protect the vulnerable—elderly and patients with pre-existing comorbidities—by providing safe and assisted isolation and care; not least now that lockdown rules start to be relaxed. The vulnerable have to receive the necessary support to stay home, isolated, until it is safe again for them to return to normal life with social and physical contacts.

1. Identify and provide uninfected caregivers who do not spread the virus. Preferentially and ideally, these people will already have had COVID19 and have cleared the infection. With blood tests that measure antibodies against SARS-CoV-2 (21) we now have the tool to identify a majority of individuals that have had and cleared the infection. In some individuals the SARS-CoV-2 antibody response may be too be too low to be detected and before tests assessing T cell immunity become available for routine use all subjects with negative tests must be assured to be non-immune and tested by RNA-based tests that can detect SARS-Co-V-2 RNA in respiratory specimens. These tests must be performed routinely and regularly in people who assist isolated people. The ability to test large numbers of individuals, rapidly, repeatedly and effectively, for the presence of the virus, and for the existence of immunity is a cornerstone of this strategy. Certification of tests should be fast-tracked, as time is of essence here.
2. Educate caregivers on how to avoid spreading the virus, including hygiene rules and provision of personal protective equipment, including respiratory protective and risk mitigation measures.
3. Establish programs for home delivery of food, medications, and other essential items, to avoid unnecessary exposure, especially of the vulnerable populations. Clear protocols for handling and cleaning the delivered goods must also be established.
4. Provide shelter for those infected, isolating them from family members, and to those who are already in the proximity of infected family members to avoid transmission from asymptomatic family members to vulnerable ones.
5. In parallel, isolation of individuals with symptoms, diagnosed cases, and their contacts should continue (22).

These measures are per se not easy to accomplish but might be more efficient than the universal lockdown that is being pursued in different countries to different degrees.

## How Can We Most Safely and Rapidly Revert to “Normal”?

Here, we face two main options and some potentially risky and tough choices.

- The prolonged complete lockdown is not sustainable for an extended period of time due to its drastic and increasing economic and societal fallouts.
  - Even if successful, universal curfews would have to be implemented over many months, with unforeseeable consequences on society in many ways. Still, there are then billions of virus-naïve people who could potentially support new outbreaks.
  - The economic collapse with mass unemployment will have deleterious effects on health, including increasing mortality also in younger age groups. As an example, the much less severe economic turbulence of 2009 was calculated to cause the death of 260,000 individuals just by cancer (5), and the negative effects on health in the developing countries was very large (6).
  - In addition, these measures will, over time, destabilize society, not only through tremendous economic losses, but also through the risk of increasing social unrest and the psychological consequences of social isolation (7, 9). Selective damage to people with vulnerable job categories, particularly in countries without adequate social network safety, will put them in desperate situations and soon left without options (8).
  - Lastly, one could argue that a functioning economy and intact supply chains will better enable us to protect the more vulnerable and limit severe outcomes. We will then have enough hospital/ICU beds to take care of those who will need them.
- Protect the vulnerable and then progressively ease overall restrictions. We must carefully consider and follow the emerging epidemiological data, especially the number of infections with severe outcome in younger individuals and those without preexisting conditions, before coming to premature conclusions. However:
  - the intensification of measures to protect the vulnerable must be implemented in priority.
  - Once these measures established, day-care centers, schools, and colleges could re-open, to care for young children whose parents are unable to provide full-time care due to essential professions to prevent them from being cared for by grandparents, who need protection, not exposure.
  - In the meantime, the general population of less vulnerable and mostly younger people should still respect physical distancing and hand hygiene standards. This includes having sufficient sanitizers provided in public places, e.g., in stores at checkout lines, or public transport. Anyone in this group who exhibits any potential COVID-19 symptoms should immediately follow the recommended self-isolation procedures and not return to society until after having been symptom-free for 2 days. Otherwise, they should live near normally, work, go to school, go to shops, and consume to prevent economic downturns.
  - Economic support from governments and banks should be provided, especially for those industries/small businesses that experience a shortfall or have been forced to close down (i.e., travel, hotels, restaurants, cruises, artists, concert halls, etc.), because the vulnerable must stay isolated. Urgent funding is also required for hospitals, laboratories, and researchers to enable the fastest possible development of diagnostic assays and new therapies.
  - Gradually then, a substantial proportion of the less endangered population will become infected by SARS-CoV-2 and develop immunity, leading to a gradual end of the epidemic (something that might already be happening in hotspots). More universal testing for active virus and anti-viral antibodies could then be used to determine when it would be time to advise the vulnerable to resume a regular and normal life again. We hypothesize that such a well-controlled shorter bubble could work in our favor, allowing resumption of societal functioning and resource generation until clinically proven treatment options for the critically ill and vaccines become available.

## Discussion

There have been several pandemics in recent decades such as the Asian Flew, the Honkong flew, The Swine flew, etc., with great losses of lives, but without the dramatic influence on societies as the present pandemic. The approach to contrast the COVID19 (or SARS-CoV-2) pandemic varies greatly among countries. Intensive testing coupled with tracking and isolation has at least so far indeed bent the curve so far in South Korea and New Zealand, possibly, also in Singapore, Hong Kong, and China (the latter with rather drastic containment measures). However, these measures have isolated subjects at risk, but have not increased immunization of the population with so called herd immunity through the transient infection of the less vulnerable. Hence, they still leave plenty of risk for re-emerging outbreaks, as increasingly reported. The strategy we propose is more sustainable in the long term, protecting the vulnerable population while we wait for herd immunity to be established, either through natural infection among the lower-risk population or a vaccine. Otherwise, society would be forced to remain closed, or return to lockdown.

**Figure 1:**
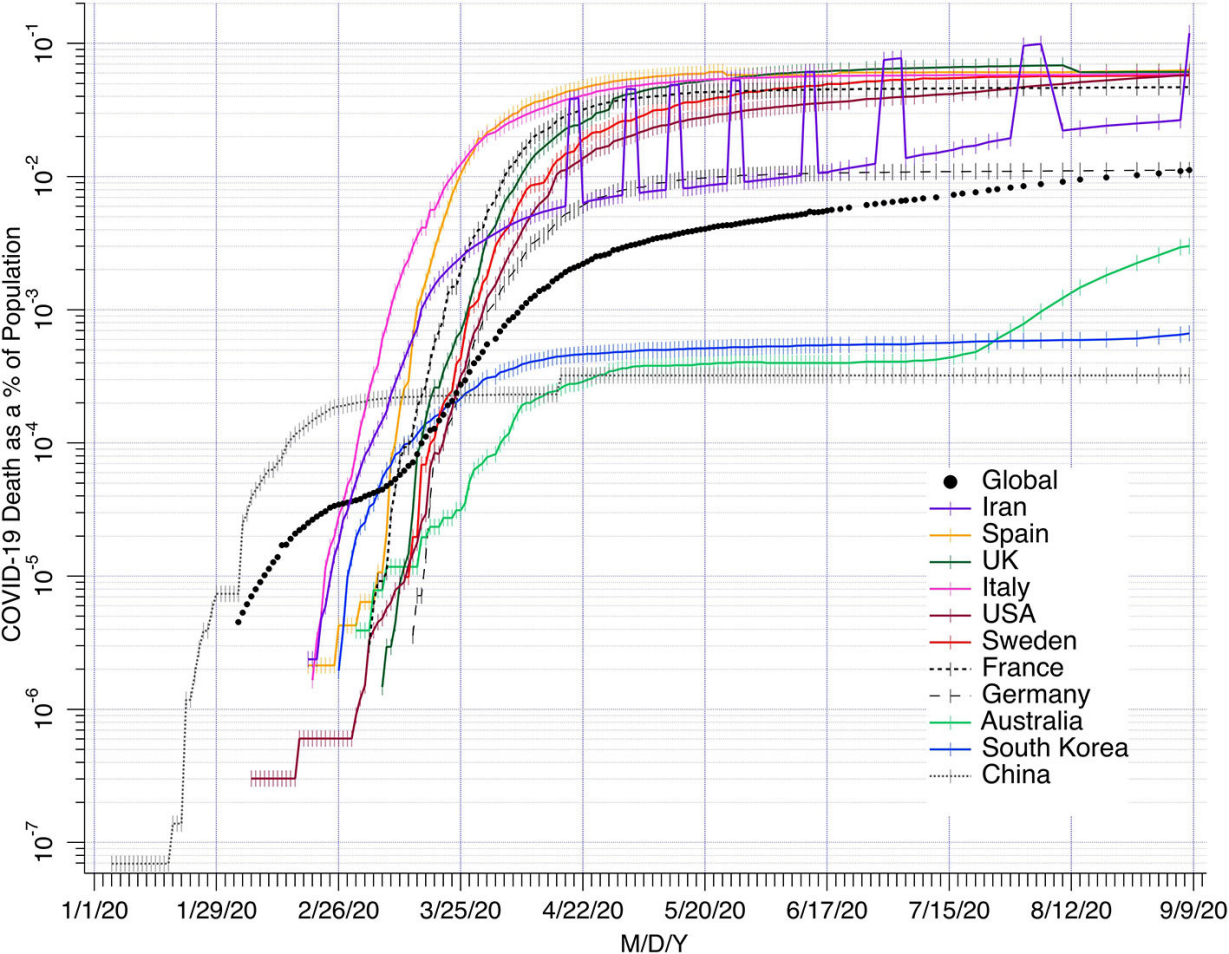
COVID19 deaths as % of the population (vertical axis). Source: Center for Disease Control and Prevention CDC USA. The seven raised data points for Iran are from ncr-iran.org.

Sweden has used a policy rather similar to our recommendations to protect vulnerable groups, without a total lock-down of the society. Day-care centers and schools, shops, and even restaurants have remained open, while maintaining hygiene and physical distancing recommendations to slow down spreading of the disease. The COVID-19 curve has been flattened enough to maintain 20–30% of ICU capacity available (23). Difficulties to keep elderly completely isolated has caused loss of many lives, but mainly among people >80 years (median age 84 years for those who have died) with co-morbidities and limited life expectancy. Yet, the death rate has remained similar or sometimes even lower than in several other European countries hit by the epidemic at the same time (Figure 1). All curves tend to a slower rate over 8 months irrespective of the degree of lockdown measures implemented. One explanation for the rather similar death rates caused by the pandemic could be that in every country there are many undocumented mild cases that spread the disease and overall a degree of herd immunity is developing. Australia, due to strict lockdown now has an increase, and the numbers of new cases with Covid19 are increasing in several European countries with previous strict lock-down.

Although some economic depression has been unavoidable because of both decreased consumption and the dependence on global economy, according to the European Union the economy is expected to be less negatively impacted in Sweden than in countries with total lock-down measures.

In conclusion, we here offer some considerations on possible paths to ease out of restrictions with a focus on protecting the vulnerable, decreasing the load on hospital, health force and caregivers, and promoting immunity in the population to reduce the risk of future epidemics.

Politicians will have to face the natural unease accompanied with releasing restrictions under such measured conditions. Still, it is key to balance restrictions with the stage of the epidemic in certain areas and with the long-term impacts that broad and severe restrictions will have. Once we emerge from the acute phase of this tragedy, we will have to divert much of our resources to preventive measures to avert future impacts of emerging viral and bacterial infections.

## Author Contributions

JL and MvH wrote the first draft. DH, DS, and MF added some references. All authors revised the manuscript and approved the final version.

## Conflict of Interest

The authors declare that the research was conducted in the absence of any commercial or financial relationships that could be construed as a potential conflict of interest.
